# Tumor Bud Classification in Colorectal Cancer Using Attention-Based Deep Multiple Instance Learning and Domain-Specific Foundation Models

**DOI:** 10.3390/cancers17071245

**Published:** 2025-04-07

**Authors:** Mesut Şeker, M. Khalid Khan Niazi, Wei Chen, Wendy L. Frankel, Metin N. Gurcan

**Affiliations:** 1 Department of Electrical and Electronics Engineering, Dicle University, Diyarbakir 21280, Turkey; 2 Department of Pathology, The Ohio State University, Columbus, OH 43210, USA; khalid.niazi@osumc.edu (M.K.K.N.); wei.chen2@osumc.edu (W.C.); wendy.frankel@osumc.edu (W.L.F.); 3 Center for Artificial Intelligence Research, Wake Forest University School of Medicine, Winston-Salem, NC 27101, USA; mgurcan@wakehealth.edu

**Keywords:** tumor budding, colorectal cancer, multi-instance learning, foundation models

## Abstract

Background/Objectives: Identifying tumor budding (TB) in colorectal cancer (CRC) is vital for better prognostic assessment as it may signify the initial stage of metastasis. Despite its importance, TB detection remains challenging due to subjectivity in manual evaluations. Identifying TBs remains difficult, especially at high magnification levels, leading to inconsistencies in prognosis. To address these issues, we propose an automated system for TB classification using deep learning. Methods: We trained a deep learning model to identify TBs through weakly supervised learning by aggregating positive and negative bags from the tumor invasive front. We assessed various foundation models for feature extraction and compared their performance. Attention heatmaps generated by attention-based multi-instance learning (ABMIL) were analyzed to verify alignment with TBs, providing insights into the interpretability of the features. The dataset includes 29 WSIs for training and 70 whole slide images (WSIs) for the hold-out test set. Results: In six-fold cross-validation, Phikon-v2 achieved the highest average AUC (0.984 ± 0.003), precision (0.876 ± 0.004), and recall (0.947 ± 0.009). Phikon-v2 again achieved the highest AUC (0.979) and precision (0.980) on the external hold-out test set. Moreover, its recall rate (0.910) was still higher than that of UNI’s (0.879). UNI exhibited a balanced performance on the hold-out test set, with an AUC of 0.960 and a precision of 0.968. CtransPath showed strong precision on the external hold-out test set (0.947) but had a slightly lower recall (0.911). Conclusions: The proposed technique enhances the accuracy of TB assessment, offering potential applications for CRC and other cancer types.

## Simple Summary

Tumor budding (TB) is a key factor in colorectal cancer (CRC) prognosis, but its detection is challenging due to subjectivity and inconsistencies in manual evaluation. To address this, we propose an automated deep learning-based TB classification system. Using weakly supervised learning, we aggregate positive and negative bags from the tumor invasive front. This study leverages histopathological foundation models for tumor budding classification. CTransPath and Phikonv2 achieved the highest AUCs on the external hold-out test set, with 0.962 and 0.979, respectively. The weakly supervised ABMIL framework highlights regions of interest in H&E-stained slides. Our approach improves TB detection accuracy, supporting its potential use in colorectal cancer prognosis and broader cancer research.

## 1. Introduction

The American Cancer Society reports that colorectal cancer (CRC) ranks as the third most prevalent cancer and is the second leading cause of cancer-related mortality in the U.S., with approximately 151,000 new cases anticipated in 2022 [1]. Detecting tumor budding (TB) in CRC is crucial, as it correlates with poorer prognoses and serves as a biomarker for more precise patient risk classification. TB signifies a form of epithelial-to-mesenchymal transition, marking the initial biological phase toward metastasis. While TB is recognized as a significant prognostic indicator in CRC, its application in routine clinical practice faces challenges due to a lack of consensus on the most effective evaluation method. Guidelines from the College of American Pathologists Cancer Protocol and the International Tumor Budding Consensus Conference recommend using hematoxylin and eosin (H&E) slides for TB assessment. Nonetheless, several studies indicate that identifying TBs on H&E slides is challenging and nearly impossible to reproduce consistently [2,3]. Manual TB detection requires meticulous examination of the tumor’s invasive edge at intermediate to high magnification powers, a process that is both time-intensive and subjective. This subjectivity in detection presents a major hurdle to efficiently employing this critical prognostic factor in CRC assessments.

In CRC, TBs are now recognized as a key prognostic marker [4,5]. TB is one of the features assessed in malignant polyps and stage II CRC to help predict a patient’s prognosis and guide the oncologist in determining the best treatment. Histologically, TB reflects the separation or budding of cancerous cells from tumor glands at the invasive edge of the tumor [6,7]. Defined as clusters of one to four tumor cells at this invasive front [8], TBs mark the initial biological step toward metastasis, following a transition from epithelial to mesenchymal characteristics [9,10]. This transition allows tumor cells to detach from the main tumor mass, spread to distant body sites, and establish metastases. Identifying TB in CRC is critical due to its association with negative outcomes and potential as a biomarker for improved risk stratification. However, the routine detection of TBs in clinical practice is limited by a lack of consensus among pathologists. Clinically, when TB is found, it will be graded as low, intermediate, or high, based on histologic evaluation of a biopsy (such as a polypectomy for a malignant polyp) or a surgical resection specimen. A high TB score is considered a high-risk feature associated with an increased likelihood of lymph node metastasis; therefore, this may impact the next step in the management of the patient. Patients with high TB may benefit from additional treatments, such as colectomy when identified in a biopsy specimen or adjuvant chemotherapy when found in a resection specimen. Unfortunately, there are currently no known preventive measures for TB. TB reflects molecular alterations that have already occurred within the tumor that promote the epithelial–mesenchymal transition, enhancing tumor cell motility and invasiveness. The College of American Pathologists (CAP) Cancer Protocol and the International Tumor Budding Consensus Conference (ITBCC) recommend assessing TB on hematoxylin and eosin (H&E) stained slides [11].

Digital pathology is pivotal in enhancing clinical oncology workflows [12,13]. The rapid evolution of artificial intelligence (AI) significantly benefits computational histopathology, offering solutions to a range of diagnostic and prognostic issues. Currently, evaluating TB demands a detailed examination of the tumor’s invasive edge at intermediate to high magnification, identifying tumor-bud hotspots, and counting buds for grading, which makes it a labor-intensive task [14]. While automated CRC diagnostic methods have advanced considerably, challenges persist in bridging the gap between pathologists’ evaluations of TB and the H&E-stained images used for training deep learning models. Effective deep learning model training relies on precise ground truth data, yet variations in annotations by pathologists introduce substantial variability, resulting in noisy annotated regions and inconsistent outcomes [15].

The field of digital pathology is advancing rapidly but faces critical challenges. These include addressing diverse and intricate clinical problems, often involving rare conditions, while dealing with a scarcity of labeled data [16]. Such limitations impede the creation of reliable AI-powered tools in the biomedical domain, where precision in probabilistic outcomes is crucial. To overcome these obstacles, foundational models in digital pathology are emerging, designed with careful consideration of the size and variety of pretraining datasets, as well as model parameters [17,18,19].

There are various machine learning-based methods for TB detection in CRC histopathology images. These studies have utilized diverse approaches to handle complex data structures and limited annotations. For instance, the Segment Anything Model (SAM), combined with a SAM-Adapter, has been adapted for the efficient semantic segmentation of TB regions [20]. In addition, the work includes innovative image registration techniques, where pan-cytokeratin images are aligned with adjacent H&E-stained images, allowing for accurate transfer of TB regions between pan-cytokeratin stain and H&E [15]. Attention-based multiple instance learning (ABMIL) has also been employed for robust TB classification, while advanced feature extraction models like CTransPath have improved classification accuracy [21]. Furthermore, generative models, such as cGAN [22] and DatasetGAN [23], have been used to generate synthetic images, address data limitations, and enhance the robustness of TB detection. Finally, ABMIL has been conducted for TB classification, utilizing SqueezeNet pretrained on ImageNet as a feature extractor to generate instance embeddings for end-to-end training to emphasize the effectiveness of transfer learning approaches in classifying TB regions [24].

Convolutional neural networks (CNNs) have also been widely utilized in TB detection tasks within CRC research, addressing objectives such as object detection, segmentation, classification, and quantification. These models are particularly suited for medical image analysis due to their ability to process spatially structured data, a fundamental characteristic of images. One of the primary advantages of CNNs is their ability to automatically extract hierarchical features across network layers, enabling efficient analysis of diverse image types [25]. Moreover, a significant focus in the field is the development of fully automated workflows that eliminate the need for manual input, thereby reducing the workload for pathologists and minimizing the risk of human error [26]. Several notable studies have advanced TB diagnosis in CRC using CNNs. These include the encoder–decoder design of SegNet [27] for TB detection and segmentation, a hybrid image analysis approach utilizing the CNN-based AlexNet [28], the VGG16 architecture for automated TB detection in CRC [29], and the development of a Faster R-CNN model by integrating a region proposal network (RPN) with the VGG16 feature extraction framework [30]. Additionally, a CNN toolbox [31] was employed to determine whether an RB candidate represented an individual TB, marking a significant step in this domain.

There are various shortcomings of the existing methods. The study conducted by Banaeeyan et al. (2020) was constrained by a small dataset (58 images from 5 WSIs) [27]. The study by Bergler et al. (2019) also relied on pan-cytokeratin staining, which may not be widely available or feasible in all settings. It used a relatively simple model (AlexNet), which may struggle with complex features [28]. In the study by Bokhorst et al. (2018), the small sample size with limited TBs (only 194 in the training set) and a low F1-score (0.36) were some of the limitations observed [29]. Lu et al. (2022) did not address interpretability or provide insights into TB localization beyond bounding boxes [30]. The study by Weis et al. (2018) relied on CNN-based tools, which may limit reproducibility in broader research communities [31]. Their small dataset consisting of only 20 slides may have reduced the model’s ability to generalize well to unseen data, as limited training samples can lead to overfitting and poor performance on external test sets. Finally, the lack of advanced CNN architectures could have hindered the detection of more complex TB patterns. Moreover, one previous research study relied solely on the CTransPath model for feature extraction, whereas our study explores various histopathology-specific foundation models, offering a broader understanding of feature extraction capabilities for TB classification. Finally, the results in [24] are impressive, yet its reliance on ImageNet—a dataset containing natural images—for transfer learning is a significant limitation in the domain of histopathology.

The current study aims to develop image analysis techniques for the automated evaluation of TB in CRC. Specifically, this involves creating methods to identify stroma (tissue) and tumor cell nuclei from H&E-stained slides. Additionally, a quantitative image analysis technique is developed to enable the automated assessment of TBs within H&E-stained CRC images. The performance of diverse feature extractor foundation models is assessed to evaluate their effectiveness in classifying TB in CRC. AB-MIL is integrated into an innovative, weakly supervised approach to extract visually interpretable features from whole slide images (WSIs). Finally, attention weights from TBs are observed to determine whether the regions highlighted by AB-MIL aligned with TBs. The methods developed in this study can be applied to other organs and cancer types, providing a more accurate, consistent, and objective TB assessment for cancer risk analysis.

## 2. Materials and Methods

### 2.1. Dataset

This study received approval from the Ohio State University Institutional Review Board (IRB 2018C0098) with a waiver for informed consent. The initial dataset includes 29 H&E-stained tissue slides, each digitized at 40× magnification using an Aperio ScanScope XT scanner (Vista, CA, USA) at a resolution of 0.061 microns per pixel squared. Board-certified pathologists (W.C. and W.F.) annotated the tumor invasive front in all cases using H&E-stained sections, separating the tumor bulk areas from the non-tumor areas. Cytokeratin AE1/3 immunohistochemical stain was performed on adjacent sections, with immunostain-highlighted TBs correlated to their counterparts on the H&E slides. This process ensured accurate TB identification, particularly in challenging cases with intense inflammation. As a result, pathologists provided manual annotations for each slide, marking TBs, tumor regions, and non-tumor areas. A total of 3344 TBs and 5573 non-tumor regions of interest (ROIs) were annotated. An additional external test set (hold-out test set) of 70 WSIs was also annotated, covering TBs, tumors, and non-tumor regions. Altogether, 11,867 TBs and 5011 non-tumor regions of interest (ROIs) were marked.

### 2.2. Method

**Figure 1:**
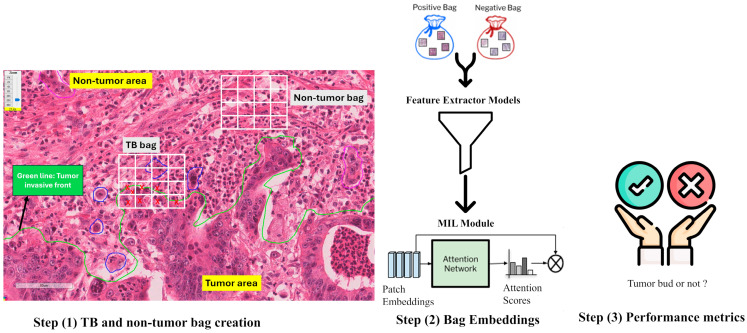
The steps followed in the proposed deep learning algorithm. First, positive/negative bags are created. Then, bag embeddings are extracted from histopathology foundation models. Finally, the MIL classifier is applied to observe performance metrics in TB classification.

The overall steps followed in the proposed methodology are composed of 3 stages: (1) tumor and non-tumor bag creation from WSIs, (2) positive and negative bag embedding creation using feature extractor models and the MIL module, and, finally, (3) performance metric evaluation for TB classification. These steps are illustrated in Figure 1.

#### 2.2.1. TB and Non-Tumor Bag Creation

**Figure 2:**
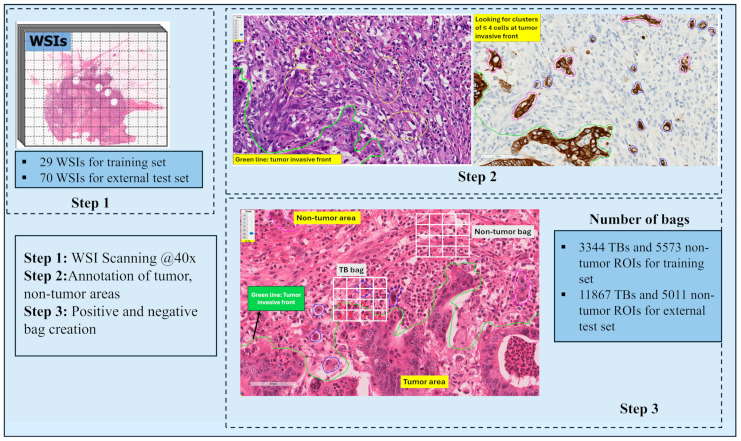
Overview of the digital pathology pipeline for tumor budding (TB) detection and analysis. Step 1: Whole slide images (WSIs) were scanned at 40× magnification, with 29 WSIs used for training and 70 WSIs for external testing. Step 2: Tumor and non-tumor regions were manually annotated, with the tumor invasive front delineated (green line). Tumor budding areas, defined as clusters of ≤4 tumor cells, were identified. Step 3: Positive (TB) and negative (non-tumor) regions of interest (ROIs) were extracted to create training and test datasets. The training set included 3344 TBs and 5573 non-tumor ROIs, while the external test set contained 11,867 TBs and 5011 non-tumor ROIs.

In order to create TB bags, each annotated TB was first enclosed in a 512 × 512-pixel region, from which smaller 96 × 96 pixel instances were cropped with a stride of 32 pixels. For non-tumor bags, 512 × 512 pixel regions were tessellated within annotated non-tumor areas and divided into 96 × 96 pixel instances. In this setup, each 512 × 512 pixel area served as a “bag”, and the 96 × 96 pixel tiles within it represented the instances. The 96 × 96 pixel size was selected based on the expected maximum size of a TB. Overall, there were 3344 TB bags and 5573 non-tumor bags. In the case of binary classification, a bag was labeled as positive if it contained at least one positive instance; otherwise, it was labeled as negative. An overview of the current WSI slides, image annotation, and bag creation can be found in Figure 2.

#### 2.2.2. Generating Feature Embeddings with Histopathology Foundation Models

We assessed the performance of four diverse feature extractor models to evaluate their effectiveness in classifying TB in CRC. These models were categorized into two groups: 3 histopathology-specific foundation models trained on publicly accessible datasets and one state-of-the-art, large-scale model developed using proprietary data. The details of these feature extractor models can be found in Table 1.

##### CTransPath

Self-supervised learning (SSL) presents a promising approach by leveraging unlabeled data to create meaningful representations that perform effectively across diverse downstream tasks, even when annotations are scarce. Wang et al. (2022) introduced a novel SSL method called semantically relevant contrastive learning (SRCL), which focuses on identifying and utilizing relevant relationships between instances to uncover more positive pairs [32]. Unlike traditional contrastive learning, which generates two views of the same instance, SRCL associates multiple positive instances with similar visual characteristics. This increases the diversity of positive samples, leading to richer and more informative representations. To implement this, they utilized a hybrid model, CTransPath, as the backbone. This model combines convolutional neural networks (CNNs) with a multi-scale Swin Transformer architecture, effectively capturing local and global features. Pretrained on a large collection of unlabeled histopathological images, CTransPath serves as a universal feature extractor tailored to the histopathology image domain. The effectiveness of their SRCL-pretrained CTransPath was validated across five downstream tasks—patch retrieval, patch classification, weakly supervised whole slide image classification, mitosis detection, and CRC gland segmentation—using nine publicly available datasets. The results demonstrated that SRCL-driven visual representations outperform state-of-the-art benchmarks in each dataset and exhibit superior robustness and transferability compared to existing SSL approaches and supervised or self-supervised ImageNet pretraining.

##### Phikon-v2

Recent developments in self-supervised learning (SSL) have greatly enhanced the ability to learn meaningful representations for histopathology, removing the necessity for pretraining on unrelated domains. In this approach, WSIs are segmented into patches and encoded using SSL-pretrained models to produce robust descriptors for a wide range of histological features. Current leading models heavily utilize the DINOv2 framework [33], an evolution of the iBOT method [34] with additional pretraining techniques designed for better scalability in terms of both data and models. Filiot et al. (2024) presented Phikon-v2 [35], an upgraded version of our earlier model, Phikon [36]. Phikon-v2 is a Vision Transformer Large (ViT-L) model, pretrained using DINOv2 on an extensive dataset of 460 million histology tiles sourced from over 55,000 publicly available slides. This dataset encompasses healthy tissues as well as more than 30 cancer types. Phikon-v2 delivers competitive results in tasks like biomarker prediction and disease classification at the WSI level, matching the performance of state-of-the-art models. The model is freely accessible on Hugging Face at “https://huggingface.co/owkin/phikon-v2 (accessed on 18 February 2025).

##### CHIEF

CHIEF employs a two-phase pretraining strategy effectively designed to capture pathology features that are beneficial for various evaluation tasks. In the first phase, self-supervised learning is utilized to derive patch-level feature representations from unlabeled data. The second phase incorporates weakly supervised learning and an attention mechanism to merge these patch-level features, producing global pathology representations for WSIs. This stage only requires WSI-level labels, allowing CHIEF to form a comprehensive understanding of pathology images based on global features [37]. The model leverages the self-supervised CTransPath backbone as its image encoder to extract histopathology feature representations. These features are then combined using attention-driven feature fusion, further refined through instance-level feature analysis and WSI-level contrastive learning. The detailed implementation can be found at “https://github.com/hms-dbmi/CHIEF (accessed on 18 February 2025)”.

##### UNI

Accurate analysis of tissue images is essential for computational pathology (CPath) applications, requiring precise characterization of histopathological structures from WSIs. However, the high resolution of WSIs and the diverse morphological features present significant obstacles, particularly in generating the large-scale annotations necessary for high-performing models. To overcome this, researchers have explored pretrained image encoders using transfer learning from natural image datasets or self-supervised learning on publicly available histopathology datasets. Despite these advancements, such methods have not been extensively optimized or tested across a broad spectrum of tissue types.

Chen et al. (2024) presented UNI, a versatile self-supervised model tailored for pathology, pretrained on a massive dataset of over 100 million images extracted from more than 100,000 diagnostic H&E-stained WSIs (>77 TB of data), representing 20 major tissue types [38]. UNI stands as the most extensive pretrained vision encoder developed specifically for histopathology. UNI was rigorously evaluated across 34 diverse CPath tasks varying in diagnostic complexity. Beyond surpassing previous state-of-the-art models, UNI introduces innovative capabilities in CPath, including resolution-independent tissue classification, few-shot slide classification using class prototypes, and generalized disease subtyping for up to 108 cancer types using the OncoTree classification framework. UNI diverges from conventional approaches by not relying on open datasets or widely used public histology slide collections, such as TCGA, CPTAC, PAIP, CAMELYON, PANDA, and others, in TCIA for pretraining, which are typically employed in benchmark development for computational pathology. UNI sets a new benchmark in large-scale unsupervised representation learning for CPath, offering data-efficient AI solutions with broad generalizability for complex diagnostic tasks and clinical workflows in anatomic pathology. The model card for UNI is available at https://huggingface.co/MahmoodLab/UNI (accessed on 18 February 2025).

#### 2.2.3. Attention-Based Multiple Instance Learning (ABMIL)

MIL is a form of weakly supervised learning that focuses on training and making predictions based on groups of instances rather than individual ones [39]. Unlike traditional supervised learning, MIL requires labels only for the group, referred to as a “bag”, rather than for each instance. This approach is particularly advantageous in pathology, where a set of patches extracted from a WSI can be treated as a bag, enabling the development of slide-level predictors without requiring detailed patch-level annotations [40]. To aggregate information from patches, attention-based pooling methods have proven effective and are widely employed in end-to-end pathology modeling [41].

The attention-based pooling mechanism dynamically learns to assign weights to embedded instances, enabling their aggregation into a bag-level feature vector. Each embedded instance is automatically assigned to a weight, and these weights are used to compute a weighted sum, producing a single bag-level representation corresponding to a slide-level embedding [42]. This bag-level embedding is then utilized for classification or regression tasks [43].

The implementation of the attention mechanism utilizes a straightforward two-layer fully connected neural network. Each instance embedding $h_{k}$ is processed through two parallel layers, *V* and *U*. A *tanh* activation is applied to the output of *V*, while a *sigmoid* activation is applied to the output of *U*. The results are then combined through an element-wise dot product and passed through another layer ($w^{T}$), which maps the fused activation into a single scalar value representing the attention weight ($a_{k}$). These interactions are summarized in Equation (1). The bag-level representation (*z*) is obtained by computing the weighted sum of the instance embeddings and their respective attention weights, as shown in Equation (2). The network parameters (*V*, *U*, and *w*) are learned automatically during the model’s training process. We employed many histopathology foundation models with frozen weights as a feature extractor to produce instance embeddings ($h_{k}$). This feature extractor was integrated with the attention mechanism and trained in an end-to-end manner.

(1) $$a_{k}=\frac{exp\left\{w^{T}\tanh\left(Vh_{k}^{T}\right)⊙sigm\left(Uh_{k}^{T}\right)\right\}}{\sum_{j=1}^{K}exp\left\{w^{T}\tanh\left(Vh_{j}^{T}\right)⊙sigm\left(Uh_{j}^{T}\right)\right\}}$$

(2) $$z=\overset{K}{\underset{k=1}{\sum }}a_{k}h_{k}$$

#### 2.2.4. Experimental Design

A six-fold cross-validation strategy was employed to evaluate the proposed ABMIL model. In each fold, 29 WSIs were split into training (10 WSIs), validation (10 WSIs), and testing (9 WSIs) sets. The training and validation sets were randomly selected in each fold, and the models were trained over 30 epochs using the Adam optimizer (β1 = 0.9, β2 = 0.999) with a learning rate of 0.0001 and a weight decay of 1 × 10^−5^. The binary cross-entropy loss was utilized for bag-level classification. Models achieving the highest AUC on the validation set during training were saved and subsequently applied to the test set. Evaluation metrics, including AUC, precision, and recall, were computed for each fold and consistently picked up for each fold. The average of these metrics across all folds was reported to assess model performance. Additionally, the best-performing fold’s model was saved and later evaluated on an external hold-out dataset of 70 WSIs to validate its generalizability.

#### 2.2.5. Rationale Behind the Data Splitting Strategy

The current approach in dividing the dataset and using an external hold-out set was carefully designed to ensure scientific rigor and practical relevance. We split the 29 whole slide images (WSIs) into training (10 WSIs), validation (10 WSIs), and testing (9 WSIs) sets while also utilizing an external hold-out dataset of 70 WSIs to validate the generalizability of our model. Two primary considerations guided this strategy. First, by adhering to the same dataset splits used in existing studies, we ensured that our work remains compatible with prior research, enabling a fair and meaningful comparison of results. This alignment with established practices is critical for benchmarking our model’s performance against existing methods and demonstrating its effectiveness in a consistent and reproducible manner. Second, the 29 WSIs were chosen for training, validation, and testing because they come with adjacent immunohistochemistry (IHC) slides, which allowed us to develop highly accurate and reliable ground truth annotations. These annotations are essential for training a robust model and ensuring the validity of our findings. In contrast, the external dataset of 70 WSIs, while valuable for testing generalizability, does not have the same level of detailed ground truth annotations. Without complementary IHC data, the annotations for the 70 WSIs are less precise, making them less suitable for training but still highly useful for validating the model’s performance on unseen data.

## 3. Results

The performance of four different foundation models was evaluated on TB histopathological datasets, with metrics averaging over six splits, and tested on an external hold-out dataset. Considering the results in Table 2, among the models, Phikon-v2 consistently demonstrated superior performance, achieving the highest average AUC (0.984 ± 0.003) across the six splits, along with notable precision (0.876 ± 0.004) and recall (0.947 ± 0.009). This indicates that Phikon-v2 is particularly effective in identifying TB in CRC. UNI also performed well, with an average AUC of 0.978 ± 0.003, precision of 0.845 ± 0.029, and recall of 0.950 ± 0.010.

Although UNI slightly lagged behind Phikon-v2 in precision, it demonstrated strong recall, making it a robust alternative. In contrast, CtransPath and CHIEF exhibited comparatively lower performance on the internal dataset, with CHIEF showing the lowest AUC (0.943 ± 0.010), precision (0.784 ± 0.052), and recall (0.882 ± 0.030). On the external hold-out test set, Phikon-v2 once again outperformed the other models, achieving the highest AUC (0.979) and precision (0.980) and demonstrating its generalizability and reliability. Moreover, its recall rate (0.910) was higher than that of UNI’s (0.879). UNI exhibited a balanced performance on the external hold-out dataset, with an AUC of 0.960 and a precision of 0.968.

CtransPath showed strong precision on the external hold-out dataset (0.947) but had a slightly lower recall (0.911), while CHIEF recorded the lowest overall metrics, indicating a limitation in handling external hold-out data effectively. These findings highlight Phikon-v2 as the most effective model for TB classification, both in internal cross-validation and on the external hold-out dataset, followed closely by UNI and CtransPath. Their strong metrics suggest these models are well-suited for histopathological applications in TB bag classification.

**Figure 3:**
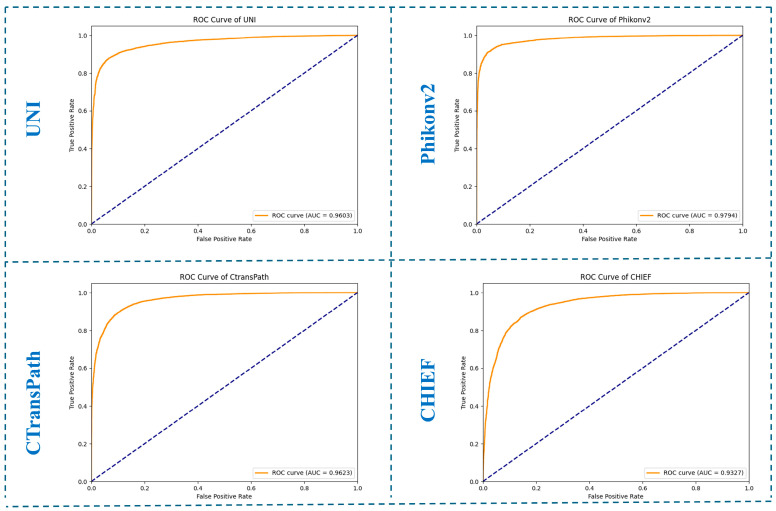
AUC-ROC curves of the histopathology foundation models within the external hold-out test set.

The Area Under the Curve (AUC) is a critical metric for evaluating the performance of classification models, particularly in imbalanced datasets. It provides a comprehensive measure of a model’s ability to distinguish between classes, with higher AUC values indicating better performance. The AUC is especially useful because it aggregates model performance across all classification thresholds, offering a robust evaluation compared to threshold-dependent metrics. In this study, we calculated AUC values to assess and compare the diagnostic performance of the histopathology foundation models (UNI, CTransPath, Phikon-v2, and CHIEF). The ROC curves, along with their corresponding AUC values for all models, are presented in Figure 3.

**Figure 4:**
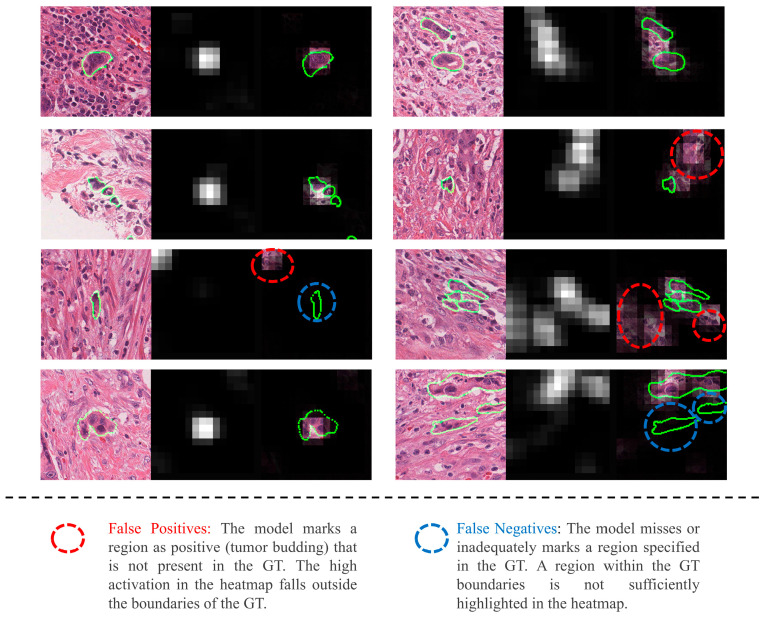
Some AB-MIL heatmaps of TB bags demonstrating successful or erroneous attention to TBs. Regions where the attention heatmap successfully aligns with the annotated TB regions are considered True Positives (TPs). Regions highlighted by the heatmap but falling outside the annotated TB regions are classified as False Positives (FPs), indicating erroneous detections. Conversely, regions within the annotated TB boundaries that are missed or inadequately highlighted by the heatmap are classified as False Negatives (FNs), representing missed detections.

We further analyzed attention weights from TB and bags to assess whether the regions highlighted by AB-MIL aligned with TBs. The outcomes of this analysis are presented in Figure 4. The first column displays pathology images with TB annotations outlined in green. The second column presents the corresponding attention heatmaps generated by the best model. The third column overlays the attention heatmap onto the original pathology image, along with the TB annotations.

## 4. Discussion

The application of foundation models for feature extraction in histopathology has shown great promise, particularly in addressing the challenges of TB classification using ABMIL downstream models. Foundation models, with their capacity to leverage diverse and large-scale datasets during pretraining, provide robust feature extraction capabilities that can be seamlessly integrated into further classification tasks. Traditional methods often rely on specialized staining techniques like pan-cytokeratin immunohistochemistry, which is not routinely employed for TB analysis in clinical practice, limiting its applicability [44]. In contrast, recent advances in AI-driven methods leverage H&E-stained slides, addressing data availability challenges and annotation variability. Approaches utilizing only H&E-stained slides offer a more feasible and scalable alternative for clinical workflows. Notably, weakly supervised approaches have shown promise in reducing the need for detailed annotations, offering solutions to inter- and intra-reader variability issues [45]. Instead, annotations can be simplified to coarse regions, allowing for efficient model training while accommodating annotation discrepancies.

ABMIL further enhances the applicability of these methods by aggregating features from bag-level encodings and identifying regions of interest, such as TB. This approach is particularly advantageous in classifying subtle histopathological features, even when annotations are sparse or inconsistent. Despite these advancements, several limitations persist. For instance, weakly supervised methods may occasionally misclassify poorly differentiated clusters (tumor cell clusters of ≥5 cells that lack gland formation) as TB due to their morphological similarities, especially without clear cellular thresholds. Additionally, current methods often adopt a binary classification framework, which does not fully capture the clinical significance of varying degrees of TB. The binary nature of many current TB classification approaches limits their clinical utility, as they focus solely on identifying the presence of TB rather than quantifying its degree. This is significant because higher TB counts correlate strongly with a poorer prognosis. Incorporating model outputs to estimate the severity or density of TB could address this limitation and enable more clinically meaningful predictions. Additionally, integrating histopathological features beyond TB into prognostic models, such as overall features of the invasive tumor front, could broaden the impact of these methods. Future work could focus on integrating probabilistic outputs from ABMIL with clinical features to stratify TB severity and predict patient prognosis. Incorporating grading systems that distinguish between high and low levels of TB may provide more clinically relevant insights. Moreover, leveraging foundation models to identify broader histopathological patterns beyond TB classification could unlock new avenues for predicting outcomes and enhancing the clinical utility of digital pathology tools.

Recent studies have employed the same dataset and methodological framework as our current work, enabling a direct comparison of results and approaches. Sajjad et al. (2024) proposed a weakly supervised deep learning method for TB classification from routine H&E-stained images without requiring detailed tissue-level annotations [21]. They introduced Bayesian Multiple Instance Learning (BMIL), which enhances generalizability and stability by incorporating multiple annotated regions during training. Using the same dataset of 29 CRC H&E-stained slides, with an average of 115 TBs per slide, their method achieved an average precision of 0.94 and recall of 0.86 in a six-fold cross-validation setup. Additionally, they set aside 70 external hold-out slides, annotated with 11,867 TBs and 5011 non-tumor ROIs, for independent evaluation. Although their results are commendable, their approach relied exclusively on the CTransPath model [32] for feature extraction, limiting the exploration of diverse feature representations.

In contrast, our study evaluates multiple histopathology-specific foundation models, providing a more comprehensive understanding of feature extraction capabilities tailored to TB classification. Similarly, Tavolara et al. (2023) utilized the same dataset and a comparable methodological approach [24]. They employed SqueezeNet, pretrained on ImageNet, as a feature extractor integrated with an attention mechanism for end-to-end training. As a baseline, they compared this with Faster R-CNN using a ResNet50 backbone. Their study achieved a precision of 0.9477 ± 0.0516, a recall of 0.9131 ± 0.0568, and an AUC of 0.9482 ± 0.0679 on the external hold-out test set. While their results are impressive, the reliance on ImageNet—a dataset containing natural images—for transfer learning is a significant limitation in histopathology. The features learned in any foundation model’s initial layers are considered very basic and universal, such as edges, curves, gradients, and textures. These low-level features are often shared across a wide range of visual tasks because they represent fundamental building blocks of image understanding. Since nuclei morphology in medical imaging is relatively simple, often characterized by basic shapes, boundaries, and textures, it is highly plausible that the model, using a pretrained network like ImageNet, might primarily rely on these initial layer features during transfer learning to make predictions. In contrast, features learned from natural images may not capture the unique characteristics of H&E-stained pathology images, potentially impacting performance. In contrast, our study bypasses this limitation by leveraging foundation models trained specifically on histopathology datasets, ensuring domain-specific feature extraction and improved performance in TB classification.

In the current study, we extended the evaluation to a broader range of histopathology-specific foundation models, highlighting the importance of tailored feature extraction for CRC TB analysis. This approach ensures more robust and generalizable results and establishes a stronger baseline for future research in this domain. In this study, we evaluated the performance of four foundational models for TB classification in histopathology datasets. Among these, Phikon-v2, UNI, and CTransPath emerged as strong performers, each model exhibiting unique strengths. Phikon-v2 achieved the highest overall metrics, likely due to its extensive training dataset (456 M patches) and the DINOv2 self-supervised learning framework. Its robust architecture (ViT-L) and diverse, multi-source training data likely contributed to its superior generalizability, reflected in its strong AUC and precision in cross-validation and external hold-out testing.

UNI also delivered competitive results, excelling in recall, which is crucial for detecting subtle features like small TBs. Its use of the same ViT-L backbone and DINOv2 framework, combined with a carefully curated 100 M-patch dataset, underscores the importance of quality over sheer dataset size in certain contexts.

CTransPath, despite having a smaller training dataset (15 M patches), demonstrated impressive precision on the external hold-out test set, highlighting the potential of its SwinT backbone and the MoCo-v3 training approach. With further fine-tuning or an expanded training dataset, CTransPath’s performance could likely match or exceed that of the top models, particularly in terms of recall and generalizability. CHIEF, while showing relatively lower performance, may benefit significantly from fine-tuning or additional training on more diverse datasets. Its results suggest potential underfitting or insufficient representation of TB features during training. The attention maps generated by our method demonstrate their capability to classify larger TBs, while smaller buds, such as single tumor cells, remain challenging to identify. To improve the effectiveness of these maps, incorporating a penalty for low attention weights within annotated regions could help refine the model’s focus and enhance its sensitivity to smaller TBs. Overall, the findings indicate that the differences in performance among Phikon-v2, UNI, and CTransPath are not substantial, with each model showcasing strengths in different areas. The results emphasize the importance of training data diversity, robust architecture, and fine-tuning in optimizing models for histopathological applications like TB classification.

A key aspect of our study was the strategic use of the 29-WSI dataset for training and validation while reserving the external dataset of 70 WSIs exclusively for testing the model’s generalizability. This approach was designed to address potential concerns about overfitting and to ensure that the model performs robustly on data from different sources or conditions. The 29-WSI dataset, though relatively small, was chosen for training due to the availability of adjacent immunohistochemistry (IHC) slides, which enabled the creation of highly accurate ground truth annotations. These annotations were critical for training a reliable model and ensuring the validity of our findings. In contrast, the external dataset of 70 WSIs, while larger and more diverse, lacks the same level of detailed annotations due to the absence of complementary IHC data. As a result, it was not suitable for training but served as an ideal testbed for evaluating the model’s generalizability. By using this external dataset for validation, we demonstrated that the model is not overfit to the 29-WSI dataset and can perform well on independent, real-world data. This approach not only strengthens the scientific rigor of our study but also provides strong evidence of the model’s robustness and practical applicability. We recognize that the 29-WSI dataset is limited in size, but the high-quality annotations it provides make it the best choice for training. By combining this with the external dataset for validation, we strike a balance between leveraging precise annotations for model development and rigorously testing generalizability on a larger, independent dataset. This strategy ensures that our study is both methodologically sound and practically relevant, addressing potential limitations while maximizing the reliability and generalizability of our results.

The dataset used in this study exhibits a mild class imbalance, with a sample ratio of 3:5 between TB and non-tumor bags. While this imbalance reflects the natural distribution of the problem we aim to solve, it raises questions about its potential impact on model training. However, the number of samples in both classes is sufficient to train a robust classifier, and retaining this imbalance ensures that the model generalizes effectively to real-world conditions. To address any potential bias arising from the imbalance, we evaluated the model using AUC-ROC, a metric well-suited for imbalanced datasets. Our results demonstrate that the model performs well across both classes, achieving balanced precision and recall, which underscores its reliability in handling the inherent imbalance of the data.

This study highlights the potential of leveraging foundation models for feature extraction and TB classification in CRC histopathology. However, there are several avenues for future research to build upon our findings. Current approaches predominantly focus on the binary classification of TB presence. Future work could explore models capable of quantifying TB severity, providing clinically relevant insights into the degree of TB and its impact on prognosis. Developing ABMIL models that generate probabilistic outputs or stratify TB severity into clinically significant categories (e.g., low, intermediate, high) could improve model interpretability and utility in clinical workflows. However, as one of the limitations in this study, the current ground truth includes areas annotated with suspected TBs, which introduces uncertainty and may require refinement for these models to achieve higher reliability. Despite our promising results, the performance metrics could be further improved by training models on larger, more diverse histopathology datasets. While this study evaluated a subset of foundation models, future research could benchmark a broader range of models, exploring how different architectures and training paradigms influence performance in histopathology tasks.

This study lays a solid foundation for TB classification, yet several unexplored avenues remain that could deepen our understanding and improve the methodology. In future work, we aim to design an algorithm for supervised contrastive MIL. This approach will leverage histopathological foundation models as encoders to generate feature embeddings, which will subsequently be used to construct multiple MIL bags for each WSI. In the first stage, feature embeddings will be aggregated into bag-level representations using an attention-based MIL mechanism. These bag-level encodings will then be trained using a supervised contrastive loss, where bag-level encodings from the same slide will act as “transformations” that attract each other in the embedding space while contrasting with encodings from other slides. This process will dynamically generate new pairs of MIL bags for each slide in every training iteration, ensuring robust representation learning. In the second stage, a classifier will be trained using cross-entropy loss to predict bag-level labels. This two-stage pipeline will enable a seamless transition from learning discriminative feature representations in the embedding space to training a classifier capable of accurate predictions at the bag level.

## 5. Conclusions

This study highlights the transformative potential of using foundation models for feature extraction and TB classification in CRC histopathology. Employing histopathology-specific models such as Phikon-v2, UNI, and CTransPath ensures domain-relevant feature extraction, making them well-suited for TB analysis in CRC. These models are designed to capture intricate histopathological patterns, leading to more accurate and reliable feature representations. Weakly supervised learning further aligns the methodology with practical clinical workflows, reducing dependency on extensive annotations and enabling the application to routine H&E-stained slides without requiring specialized staining techniques. Comprehensive evaluations across cross-validation and external hold-out test datasets enhance the robustness and generalizability of the findings. Additionally, the integration of attention mechanisms within ABMIL allows the identification of key regions of interest, improving both interpretability and clinical relevance in classifying TB.

## Figures and Tables

**Table 1:** A comparative evaluation of foundation models tailored for histopathology as feature extractors is presented. The models are divided into two categories: (blue) those trained on publicly accessible datasets and (purple) large-scale models trained on proprietary datasets. The table highlights key distinctions, including CNN vs. self-attention (SA) mechanisms, backbone architectures, the number of parameters, and specifics about the training datasets, such as their sources, tissue types, and the number of images. It is important to note that all WSIs are H&E stained.

Model	Operation Type	Backbone Architecture	Parameters	Training Type	Training Data Origin	Tissue Types	Magnification	WSIs (Patches)
**CTransPath**	CNN-SA	SwinT	27.5 M	MoCO-v3	TCGA, PAIP	Cancer Normal	20×	32,200 (15 M)
**Phikon-v2**	SA	Vit-L	307 M	DINOv2	TCGA, CPTAC, GTeX, Multiple Public	Cancer Normal	20×	58,359 (456 M)
**CHIEF**	CNN-SA (vision encoder) SA (text encoder)	SwinT (vision encoder) Transformer (CLIP text encoder)	27.5 M (vision encoder) ~63 M (text encoder)	Multiple	TCGA, CPTAC	19 anatomical sites	10×	60,530 (15 M)
**UNI**	SA	ViT-L	307 M	DINOv2	Internal-GTeX	Cancer Normal	20×	100,426 (100 M)

**Table 2:** Results of TB classification methods on the test set and the external hold-out test set. Average AUC, precision, and recall, along with standard errors from six-fold cross-validation models, are reported during training. The best model derived from an initial set of slides was saved, and the resulting model was then applied to an external hold-out test set.

Datasets	Histopathology Foundation Models	AUC	Precision	Recall
Average test metrics over six splits	UNI	0.978 ± 0.003	0.845 ± 0.029	0.950 ± 0.010
	CtransPath	0.970 ± 0.005	0.828 ± 0.005	0.919 ± 0.017
	Phikon-v2	0.984 ± 0.003	0.876 ± 0.004	0.947 ± 0.009
	CHIEF	0.943 ± 0.010	0.784 ± 0.052	0.882 ± 0.030
External hold-out test set metrics	UNI	0.960	0.968	0.879
	CtransPath	0.962	0.947	0.911
	Phikonv2	0.979	0.980	0.910
	CHIEF	0.932	0.908	0.922

## Data Availability

The datasets presented in this article are not readily available because the data are part of an ongoing study.

## Abbreviations

The following abbreviations are used in this manuscript:

TB	tumor budding
CRC	colorectal cancer
ABMIL	attention-based multiple instance learning
CAP	College of American Pathologists
ITBCC	International Tumor Budding Consensus Conference
AI	artificial intelligence
SAM	Segment Anything Model
CNN	convolutional neural network
ROI	region of interest
WSI	whole slide image
SSL	self-supervised learning
SRCL	semantically relevant contrastive learning
CPath	computational pathology
SA	self-attention
BMIL	Bayesian Multiple Instance Learning
